# Impact of AADAC gene expression on prognosis in patients with Borrmann type III advanced gastric cancer

**DOI:** 10.1186/s12885-022-09594-1

**Published:** 2022-06-09

**Authors:** Yufei Wang, Tianyi Fang, Yimin Wang, Xin Yin, Lei Zhang, Xinghai Zhang, Daoxu Zhang, Yao Zhang, Xibo Wang, Hao Wang, Yingwei Xue

**Affiliations:** 1 Department of Gastroenterological Surgery, Harbin Medical University Cancer Hospital, Harbin Medical University, Harbin, 150081 China; 2 Department of Pathology, Harbin Medical University, Harbin, China

**Keywords:** Gastric cancer, AADAC, Borrmann type III, Prognosis

## Abstract

**Background:**

The prognosis of Borrmann type III advanced gastric cancer (AGC) is known to vary significantly among patients. This study aimed to determine which differentially expressed genes (DEGs) are directly related to the survival time of Borrmann type III AGC patients and to construct a prognostic model.

**Methods:**

We selected 25 patients with Borrmann type III AGC who underwent radical gastrectomy. According to the difference in overall survival (OS), the patients were divided into group A (OS<1 year, *n*=11) and group B (OS>3 years, *n*=14). DEGs related to survival time in patients with Borrmann type III AGC were determined by mRNA sequencing. The prognosis and functional differences of DEGs in different populations were determined by The Cancer Genome Atlas (TCGA) and Gene Expression Omnibus (GEO) public databases. The expression of mRNA and protein in cell lines was detected by quantitative real-time reverse-transcription polymerase chain reaction (qRT-PCR) and Western blot (WB). Immunohistochemical (IHC) staining was used to detect protein expression in the paraffin-embedded tissues of 152 patients with Borrmann type III AGC who underwent radical gastrectomy. After survival analysis, nomograms were constructed to predict the prognosis of patients with Borrmann type III AGC.

**Results:**

Arylacetamide deacetylase (AADAC) is a survival-related DEG in patients with Borrmann type III AGC. The higher the expression level of its mRNA and protein is, the better the prognosis of patients. Bioinformatics analysis found that AADAC showed significant differences in prognosis and function in European and American populations and Asian populations. In addition, the mRNA and protein expression levels of AADAC were high in differentiated gastric cancer (GC) cells. We also found that AADAC was an independent prognostic factor for patients with Borrmann type III AGC, and its high expression was significantly correlated with better OS and disease-free survival (DFS). Nomogram models of AADAC expression level combined with clinicopathological features can be used to predict the OS and DFS of Borrmann type III AGC.

**Conclusion:**

AADAC can be used as a biomarker to predict the prognosis of Borrmann type III AGC and has the potential to become a new therapeutic target for GC.

**Supplementary Information:**

The online version contains supplementary material available at 10.1186/s12885-022-09594-1.

## Background

Gastric cancer (GC) is the sixth most common cancer and the third leading cause of cancer-related mortality, with more than 865,000 deaths every year [1]. In China, more than 80% of patients are diagnosed with advanced GC (AGC), while more than 60% of patients are diagnosed with early GC (EGC) in Japan and South Korea [2, 3]. To evaluate the prognosis of AGC patients, Borrmann classifications, which were proposed by pathologists according to macroscopic tumor characteristics, have been widely used in clinical practice [4]. With the development of research, Borrmann type III is not only the most common macroscopic type but also has different prognoses according to clinicopathological features such as tumor diameter and vascular infiltration [5–8]. However, clinicopathological features alone cannot provide comprehensive and effective prognostic information for GC patients. To evaluate Borrmann III AGC patient prognosis more objectively and accurately, we expect to screen prognostic differential genes according to their different survival times.

Our study found that arylacetamide deacetylase (AADAC) was a differentially expressed gene significantly related to the survival time of Borrmann type III AGC by mRNA sequencing. AADAC shows intracellular triglyceride lipase activity in the liver, increases intracellular fatty acid levels by hydrolyzing triglycerides, and participates in the metabolic activation of aromatic amine carcinogens [9, 10]. Although previous studies have proven that high mRNA expression of AADAC is an adverse prognostic factor for GC through public databases [11, 12], the expression of AADAC mRNA and its encoded protein in Borrmann type III AGC is not clear. In view of the important role of AADAC in lipid metabolism and chemical carcinogen metabolism, it is worth exploring the significance of its expression for the prognosis of Borrmann type III AGC patients.

In this study, we verified the effect of AADAC on the prognosis of GC patients in different populations through public databases and analyzed the mRNA and protein expression of AADAC in GC cell lines with different degrees of malignancy. In addition, we obtained tumor tissue samples from 152 patients with Borrmann type III AGC who underwent radical gastrectomy at Harbin Medical University (HMU) Cancer Hospital for immunohistochemical staining. The relationship between the protein expression level of AADAC and clinicopathological factors and prognoses in patients with GC was analyzed to construct a nomogram to predict disease prognosis in patients with Borrmann type III AGC.

## Methods

### Patients and specimens

We obtained 25 pairs of fresh frozen GC tissues and paired adjacent normal gastric mucosa tissues from Borrmann type III AGC patients who underwent radical gastrectomy [13] at the HMU Cancer Hospital for mRNA sequencing to construct the HMU-GC cohort. All separated tissues were frozen immediately in liquid nitrogen and then stored at -80℃ until RNA isolation. RNA isolation, library construction, and mRNA sequencing were performed by Novogene (Beijing, China). The data were deposited in the Gene Expression Omnibus (GEO) repository (PRJNA718168). Paraffin-embedded GC tissues from 152 patients with Borrmann type III AGC who underwent radical gastrectomy at HMU Cancer Hospital were used for immunohistochemical staining. All samples were collected after written informed consent was obtained from the patients. The study was approved by the HMU Cancer Hospital Institutional Review Board. The diagnosis of GC was based on tissue samples obtained during gastroscopy and confirmation by pathologists through examination of postoperative tissue specimens. During hospitalization, patients underwent routine preoperative examinations, including magnetic resonance imaging/gastric computed tomography (CT), abdominal ultrasonography, chest radiography, electrocardiography, hematological examination and tumor marker examination. Some patients underwent positron emission tomography (PET)/CT if necessary. Patients were followed up until the date of death, or for 5 years, whichever came first.

The exclusion criteria were as follows: (1) preoperative chemotherapy; (2) severe heart disease; (3) remnant gastric cancer; (4) postoperative confirmation of stage IV disease; (5) history of partial resection; (6) history of other malignant tumors; (7) esophagogastric junction tumor; and (8) endocrine carcinoma.

AGC is defined as a tumor that invades the muscular propria (T2) or deeper regardless of the status of lymph node metastasis [14]. The Borrmann classification was confirmed by postoperative macroscopic pathological examination, defined as follows: type I (polypoid tumors, sharply demarcated from the surrounding mucosa); type I (ulcerated tumors with raised margins surrounded by a thickened gastric wall with clear margins); type III (ulcerated tumors with raised margins, surrounded by a thickened gastric wall without clear margins); type IV (tumors without marked ulceration or raised margins, the gastric wall is thickened and indurated and the margin is unclear); and type V (unclassifiable) [14].

Postoperative chemotherapy regimens were based on the National Comprehensive Cancer Network Clinical Practice Guidelines in Oncology [15]. Oxaliplatin +capecitabine (XELOX) or oxaliplatin +S-1 (SOX) are the main treatment options for patients with stage II or III GC. To ensure the accuracy of the study, we included 77 patients who received complete postoperative chemotherapy at our institution. We did not include patients who did not undergo treatment at our institution, or who returned to the local hospital after surgery and had incomplete chemotherapy records.

### Clinicopathological data

Clinicopathological data of the patients were saved in the Gastric Cancer Information Management System v1.2 of the Harbin Medical University Cancer Hospital (Copyright No. 2013SR087424, *http: *www.sgihmu.com), including sex, age, body mass index (BMI), tumor diameter, tumor location, histological type, metastatic lymph node ratio (mLNR), pT stage, pN stage, Borrmann type, vascular infiltration, nerve infiltration, postoperative chemotherapy and laboratory examination. The mLNR was defined as the ratio of the number of metastatic lymph nodes to the number of examined lymph nodes. pTNM stage was consistent with the eighth edition of the American Joint Commission on Cancer (AJCC). Tumor marker or radiographic examinations (CT) and other adjuvant examinations were performed on all patients every 3 months postoperatively. In addition, PET/ CT examinations were performed as needed. Recurrence and metastasis can be determined by medical history, physical examination, imaging evaluation, cytologic examination, or tissue biopsy.

### Bioinformatic analysis

We included the TCGA-STAD dataset in The Cancer Genome Atlas (TCGA) database [16] and the GSE15459 dataset in the GEO database [17] into this study for verification. The TCGA-STAD dataset includes the mRNA sequencing and clinical data of 415 GC samples (mainly European and American populations). The GSE15459 dataset includes the mRNA sequencing and clinical data of 192 GC samples (mainly Asian population). Log-rank and Kaplan–Meier methods were used to analyze the survival curves of the TCGA-STAD dataset and GSE15459 dataset. AADAC-related genes were screened through R2: Genomics Analysis and Visualization Platform [18], Kyoto Encyclopedia of Genes and Genomes (KEGG) [19] and Gene Ontology (GO) analyses were performed. KEGG and GO pathway enrichment analyses were used for functional annotation of gene set. KEGG and GO analyses in the HMU-GC cohort were performed using the OmicShare tools, a free online platform for data analysis [20]. Protein-protein interaction (PPI) networks were performed using the STRING program [21]. The Student’s t-test was used to analyze the AADAC mRNA expression level among different clinicopathological features of GC patients in the GSE15459 dataset.

### Cell culture

The gastric epithelial cell line GES-1 and GC cell lines (AGS, BGC-823, HGC-27, MKN-28 and KATO III) were provided by Procell Life Science & Technology Co., Ltd. (Wuhan, China). AGS cells were cultured in Ham’s F-12 (Procell, CN), KATO III cells were cultured in IMDM (Procell, CN), and other cells were cultured in RPMI-1640 (Procell, CN). All culture media were supplemented with 10% fetal bovine serum and 1% penicillin/streptomycin solution. All cells were cultured at 37 °C with 5% CO^2^.

### RNA Isolation and Quantitative Real-Time PCR (qRT-PCR)

Total RNA was extracted from each cell line using the TRIzol reagent (Invitrogen, USA). First-strand cDNA was generated from total RNA using oligo-dT primers and reverse transcriptase (Takara, Japan). qRT-PCR was conducted using QuantiTect SYBR Green PCR Master Mix (Takara, Japan) and specific primers in a LightCycler 96 Real-time PCR Cycler (Roche, Switzerland). GAPDH was detected in each experimental sample as an endogenous control. All the reactions were run in triplicate. The relative RNA levels of AADAC in cell lines were calculated by using the 2−ΔΔCt method. Oligonucleotide sequences of the primer sets used were as follows: AADAC (forward: 5′-TCGCTGTACCTTCTGATTG-3′, reverse: 5′-TCTGTCTGCTGTCCATCT-3′) and GAPDH (forward: 5′-GACCTGACCTGCCGTCTA-3′, reverse: 5′-AGGAGTGGGTGTCGCTGT-3′).

### Western Blot (WB)

Total proteins from cell lines were extracted with RIPA lysis buffer containing proteinase inhibitor. An equal amount (30 μg) of protein sample was separated on 12% sodium dodecyl sulfate-polyacrylamide gels (SDS-PAGE) and transferred to polyvinylidene fluoride (PVDF) membranes (Millipore, USA). The membranes were then blocked with 5% nonfat dry milk in Tris-buffered saline (TBS)/0.1% Tween 20 for 1 h at room temperature. Membranes were incubated with anti-AADAC (1:1000, A10365, ABclonal, CN) and anti-GAPDH (1:1000, A19056, ABclonal, CN) primary antibodies overnight at 4 °C. The next day, membranes were washed and incubated with horseradish peroxidase-conjugated secondary antibody. Proteins were visualized using Meilunbio fg super sensitive ECL luminescence reagent (Meiluribio, CN).

### Immunohistochemistry

Formalin-fixed and paraffin-embedded sections from 152 Borrmann type III AGC patients were dewaxed in xylene and ethanol. The sections were then cleaned in distilled water. EDTA Antigen Retrieval Solution was used to pretreat the sections at pH 8.0 for 3 min at 120 °C in a pressure cooker, and endogenous peroxidase was inhibited by 3% H^2^O^2^ in PBS for 10 min. The nonspecific actions of the sections were also blocked by goat serum (Boster, USA) for 1 h at room temperature. The sections were then incubated with the primary antibody overnight at 4 °C, followed by incubation with the secondary antibody for 30 min at 37 °C. AADAC (A10365, 1:100; ABclonal, CN) was used as the primary antibody, and goat anti-rabbit IgG was used as the secondary antibody. The chromogenic reaction was performed via diaminobenzidine (DAB) staining. Image-Pro Plus software version 6.2 (Media Cybernetics, USA) was used to measure staining intensity. Three independent pathologists blindly examined all specimens based on the percentage of positive cell membranes stained. To minimize the heterogeneity of immune cell distribution, a series of optimal experimental processes was performed to reduce deviation. Without knowing the patients’ identities, the pathologists carefully examined H&E staining of multiple wax blocks from the same patient sample before the experiment. The most representative blocks covering multiple heterogeneous regions were selected to prepare tissue sections for the experiment using the same criteria. To minimize the effect of spatial heterogeneity, three images of representative fields at ×200 magnification were randomly captured in each cancer tissue. The results were quantified as the positive area/total area of immune markers, and 5.0% was defined as the cutoff value. As patients with preoperative chemotherapy and preoperative radiotherapy were excluded, the effects of chemotherapy drugs and radiation on tumor cells were absent.

### Statistical methods

Overall survival (OS) was defined as the time from surgery to death or the last follow-up. Disease-free survival (DFS) was defined as the time from surgery to recurrence/death due to disease progression or the last follow-up. OS / DFS is shown as the mean and 95% confidence intervals (CIs). The log-rank test and the Kaplan–Meier method were used to analyze survival curves. The chi-square test was used to analyze the association between AADAC expression and clinicopathological factors. Univariate and multivariate analyses based on the Cox proportional hazards regression model were used to analyze the independent risk factors for prognosis. In the univariate and multivariate analyses, age, BMI, carcinoembryonic antigen (CEA), carbohydrate antigen 19-9 (CA19-9) and mLNR were defined as continuous variables, and other clinicopathological factors were defined as categorical variables. Hazard ratios (HRs) and 95% CIs were estimated for each factor. The Student’s t-test was used to analyze AADAC mRNA expression levels, and boxplots were drawn by GraphPad Prism 8. The nomogram models were drawn through the R studio by “SvyNom” and “rms” packages. The prognostic accuracy of nomogram models was investigated by receiver operating characteristic (ROC) analysis. SPSS version 25.0 (SPSS Inc., Chicago, IL, USA) was used for analysis, and *P* < 0.05 was considered statistically significant.

## Results

### mRNA Sequencing

**Fig. 1:**
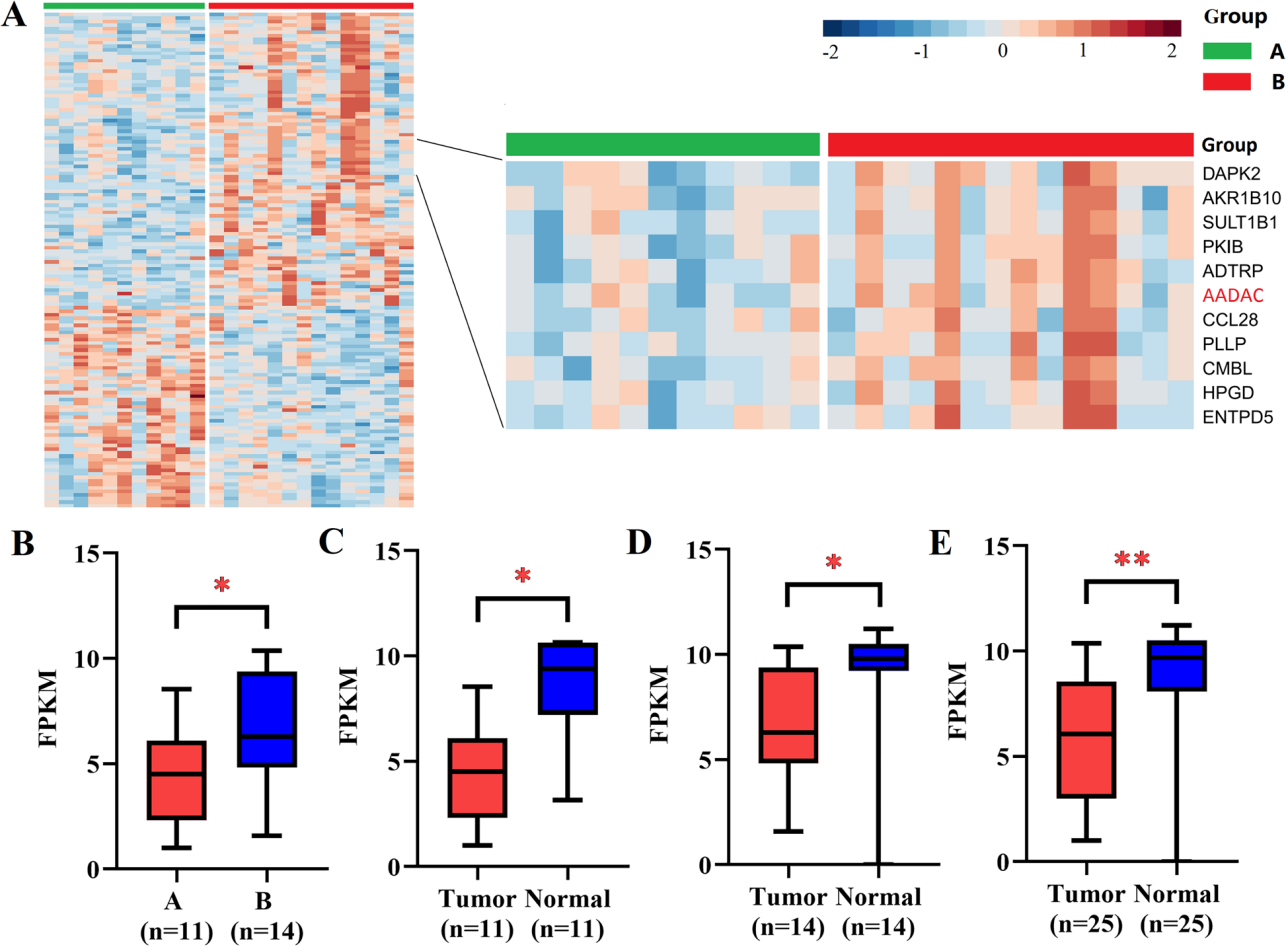
The results of mRNA sequencing. **a** Differentially expressed mRNAs in the A group (OS<1 year) and B group (OS>3 years) by mRNA sequencing. AADAC is shown in red bold font on the right. **b** The expression of AADAC in tumor tissues of group A and group B. **c** The expression of AADAC in tumor and normal tissues of all patients. **d** The expression of AADAC in tumor and normal tissues of group A. **e** The expression of AADAC in tumor and normal tissues of group B. (*P<0.05; **P<0.001.)

In this study, 25 patients with Borrmann type III AGC in the HMU-GC cohort were divided into A and B groups based on OS. The OS of patients in group A was less than 1 year (*n*=11) and that in group B was more than 3 years (*n*=14). Except for OS, the two groups of patients had no significant differences in other clinicopathological features (Supplementary file 1). Through mRNA sequencing, it was found that the AADAC gene was differentially expressed in patients in the two groups (Fig. 1a). The expression of AADAC mRNA in group A patients was significantly lower than that in group B patients (median FPKM: 4.524 vs. 6.286, *P* = 0.021) (Fig. 1b). In the 25 patients, the expression of AADAC mRNA in GC tissues was significantly lower than that in paired adjacent normal tissues (median FPKM: 6.066 vs. 9.690, *P* < 0.001) (Fig. 1c). In addition, the expression of AADAC mRNA in GC tissues of patients in groups A and B was significantly lower than that in paired adjacent normal tissues (*P* = 0.002 and *P* = 0.005) (Fig. 1d, e).

### Bioinformatics of AADAC

**Fig. 2:**
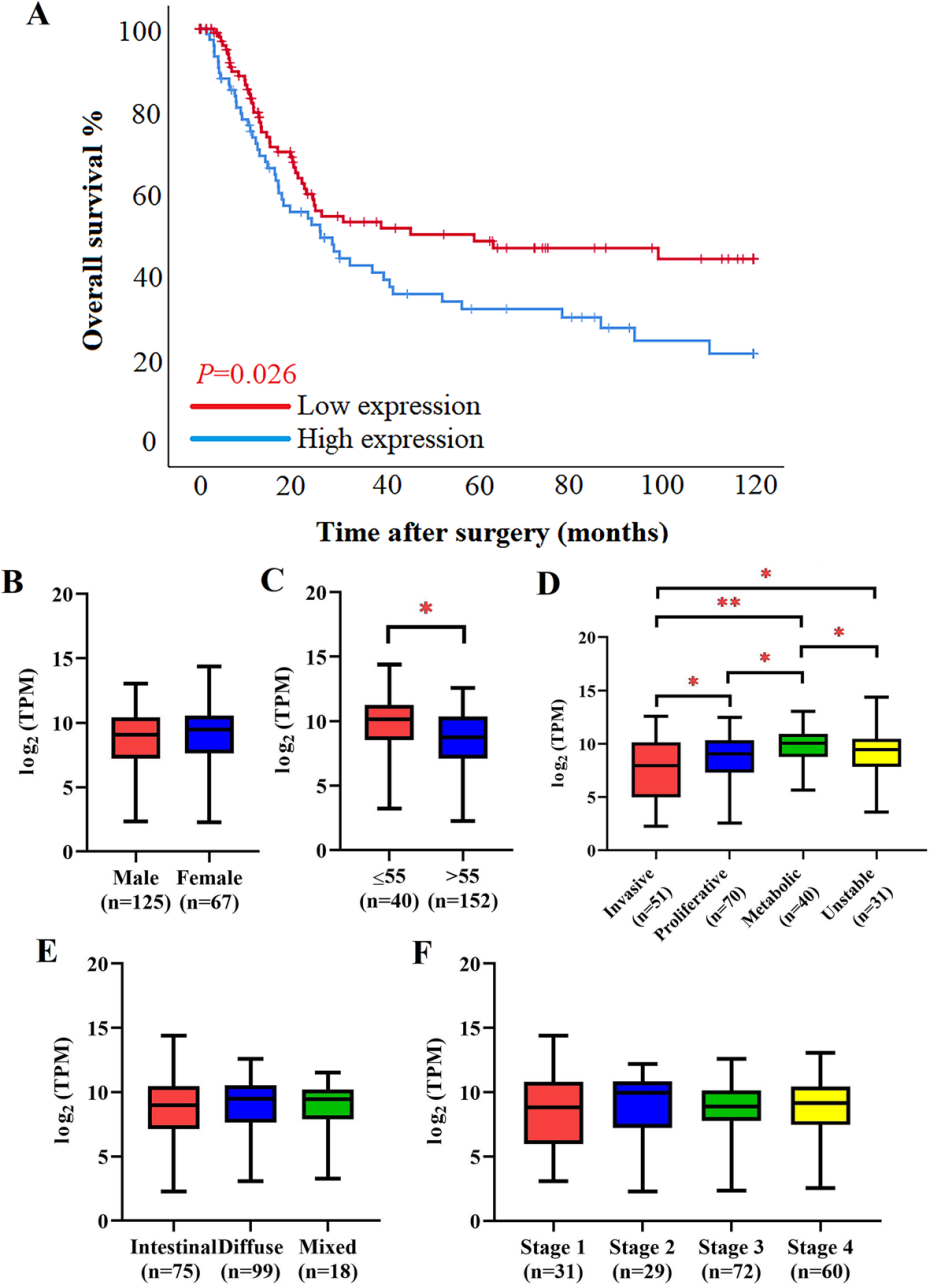
Bioinformatics of AADAC. **a** The expression of AADAC in the GSE15459 dataset. **b** The expression of AADAC based on sex in the GSE15459 dataset. **c** The expression of AADAC based on ages in the GSE15459 dataset. **d** The expression of AADAC based on subtypes in the GSE15459 dataset. **e** The expression of AADAC based on Lauren classification in the GSE15459 dataset. **f** The expression of AADAC based on pTNM in the GSE15459 dataset. (*P<0.05; **P<0.001.)

In the TCGA-STAD dataset, GC patients with high expression of AADAC had worse OS (OS: 33.42 months vs. 60.39 months, *P* = 0.003) (Supplementary file 2). In the GSE15459 dataset, GC patients with high expression of AADAC had better OS (OS: 66.31 months vs. 49.05 months, *P* = 0.026) (Fig. 2a). The tissue samples of 192 GC patients in the GSE15459 dataset showed that the mRNA of AADAC was differentially expressed in different ages (*P* = 0.002) and subtypes (*P* < 0.001). The mRNA expression of AADAC was higher in GC patients aged <55 years and GC patients with the metabolic subtype. The mRNA expression of AADAC was not significantly different by sex, Lauren type or pTNM stage (*P* > 0.05) (Fig. 2b-f).

### KEGG and GO Analysis of AADAC-related Genes

In the TCGA-STAD dataset, we found that 3224 genes were significantly related to AADAC (Table 1). By KEGG analysis, when AADAC was overexpressed, a total of 1205 genes participated in 36 signaling pathways that may play an important role in the biofilm synthesis and proliferation of tumor cells, such as glycerophospholipid metabolism, fatty acid degradation, fat digestion and absorption, tyrosine metabolism and phenylalanine metabolism (Table 2). GO analysis showed that AADAC and its related genes mainly participated in cellular lipid metabolic process, endomembrane system, cell migration, cytoskeletal organization and regulation of cell proliferation and may affect cell biofilm synthesis (Table 3). In the GSE15459 dataset, we found that 3383 genes were significantly related to AADAC (Table 1). By KEGG analysis, when AADAC was overexpressed, a total of 1196 genes participated in 42 signaling pathways that may play an important role in the degradation of chemical carcinogens and inhibit cell migration, such as chemical carcinogenesis, metabolism of xenobiotics by cytochrome P450, tight junctions and peroxisome (Table 2). GO analysis showed that AADAC and its related genes mainly participated in xenobiotic metabolic process, cell-cell adhesion, endomembrane system, maintenance of gastrointestinal epithelium and retinol dehydrogenase activity, which may affect the degradation of chemical carcinogens and intercellular adhesion (Table 3).

**Table 1:** AADAC-related genes in TCGA-STAD and GSE15459 (partial list)

TCGA-STAD						GSE15459					
Positive correlation			Negative correlation			Positive correlation			Negative correlation		
Gene	*R* value	*P* value	Gene	R value	*P* value	Gene	R value	*P* value	Gene	R value	*P* value
ATP13A4	0.524	1.31e-26	RAD23B	-0.318	2.34e-9	GSTA1	0.685	6.16e-24	KCTD20	-0.488	6.07e-11
CYP2C18	0.518	5.43e-26	SLC7A5	-0.318	2.39e-9	CTSE	0.675	4.19e-23	EHBP1	-0.464	7.40e-10
AADACP1	0.511	3.14e-25	PHF19	-0.297	3.82e-8	CYP2C18	0.674	4.46e-23	CAMSAP1	-0.451	2.84e-9
ARL14	0.497	1.19e-23	RFC2	-0.295	4.86e-8	AKR1B10	0.665	2.58e-22	CBX1	-0.445	5.10e-9
KALRN	0.496	1.25e-23	CIZ1	-0.290	9.04e-8	ADH1C	0.657	1.35e-21	CDYL	-0.435	1.32e-8
AKR1B10	0.492	3.28e-23	SET	-0.290	9.12e-8	TFF1	0.657	1.18e-21	RBFOX2	-0.430	1.97e-8
SMIM24	0.489	6.80e-23	HMGB3	-0.289	1.03e-7	SLC9A2	0.650	4.22e-21	PENK	-0.425	3.08e-8
PDZD3	0.486	1.17e-22	CEL	-0.287	1.20e-7	CYP2C9	0.645	1.22e-20	SNAP47	-0.424	3.45e-8
CTSE	0.482	3.31e-22	MZT1	-0.287	1.27e-7	BCL2L14	0.644	1.35e-20	MMD	-0.419	5.44e-8
CYP2C19	0.478	7.58e-22	CDCA4	-0.283	1.92e-7	VSIG1	0.641	2.21e-20	BOLA3-AS1	-0.415	7.69e-8

Statistically significant *P* values are in bold (*P*<0.05)

**Table 2:** Kyoto Encyclopedia of Genes and Genomes (KEGG) analysis of AADAC-related genes in patients with gastric cancer (partial list)

**TCGA-STAD**			**GSE15459**		
**Biological process**	***P* value**	**Related genes (partial list)**	**Biological process**	***P* value**	**Related genes (partial list)**
Glycerophospholipid metabolism	**0.02**	ACHE, AGPAT9, CDS1, CHPT1, ETNK1	Chemical carcinogenesis	**<0.001**	ADH1A, AKR1C2, CBR1, CYP2C18, GSTA1
Fatty acid degradation	**<0.001**	ACADL, ACOX1, ADH1A, CPT2, CYP4A11	Retinol metabolism	**<0.001**	CYP3A5, DGAT1, DHRS3, RDH12, RETSAT
Fat digestion and absorption	**<0.001**	ABCG5, APOA1, CD36, DGAT2, FABP1	Metabolism of xenobiotics by cytochrome P450	**<0.001**	CYP2C9, GSTA1, MGST2, UGT1A6
Tyrosine metabolism	**<0.001**	ADH1A, COMT, DDC, HGD, TYRP1	Tight junction	**<0.001**	CTTN, EPB41, F11R, LLGL2, MAGI1
Phenylalanine metabolism	**<0.001**	ALDH3A1, DDC, , HPD, MAOA, MAOB	Peroxisome	**<0.001**	ACSL3, CROT, DECR2, EHHADH, FAR1

Statistically significant *P* values are in bold (*P*<0.05)

**Table 3:** Gene Ontology (GO) analysis of AADAC-related genes in patients with gastric cancer (partial list)

TCGA-STAD				GSE15459				
GO path number	Biological process	Gene number	*P* value	GO path number	Biological process	Gene number	*P* value	
44255	Cellular lipid metabolic process	271	<0.001	6805	Xenobiotic metabolic process	42		<0.001
12505	Endomembrane system	808	<0.001	98609	Cell-Cell adhesion	182		<0.001
16477	Cell migration	259	<0.001	44242	Endomembrane system	638		<0.001
7010	Cytoskeleton organization	244	<0.001	30277	Maintenance of gastrointestinal epithelium	11		<0.001
42127	Regulation of cell proliferation	294	<0.001	4745	Retinol dehydrogenase activity	10		<0.001

Statistically significant *P* values are in bold (*P*<0.05)

**Fig. 3:**
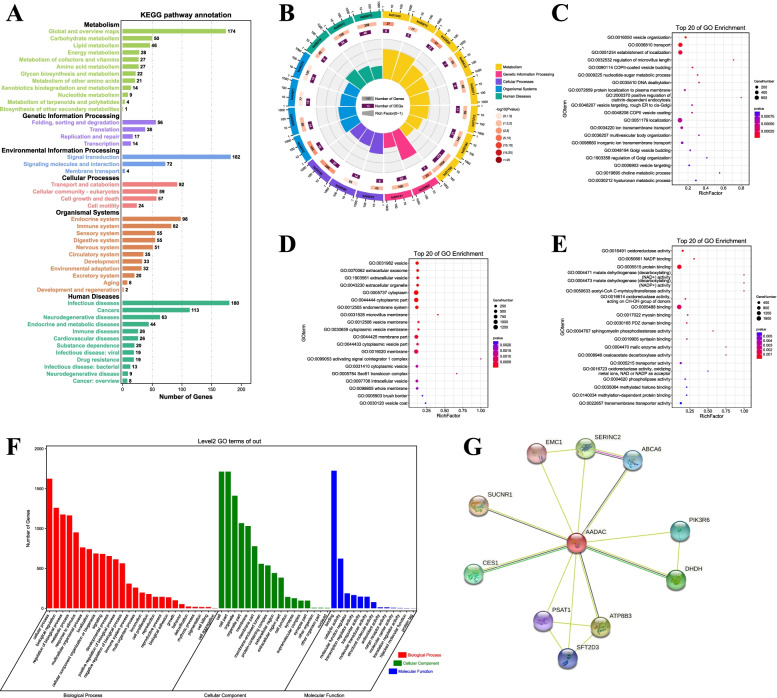
The role of AADAC in gastric cancer. **a-b** Kyoto Encyclopedia of Genes and Genomes (KEGG) pathway enrichment analyses in the HMU-GC cohort. **c-f** Gene Ontology (GO) pathway enrichment analyses in the HMU-GC cohort. **g** The interaction network of the AADAC-correlated genes in GC (STRING)

In the HMU-GC cohort, KEGG and GO pathway enrichment analyses were used for functional annotation of gene set. By KEGG analysis, when AADAC was overexpressed, AADAC and its related genes mainly participated in carbohydrate metabolism, lipid metabolism, energy metabolism, xenobiotics biodegradation and metabolism, and signal transduction (Fig. 3a, b). GO analysis showed that AADAC and its related genes mainly played roles in organelle and membrane, participated in biological processes such as biological regulation and metabolic processes, and bring into play molecular function such as binding, catalytic activity and molecular function regulator (Fig. 3c-f). In addition, we analyzed the PPI networks of AADAC by using the STRING program (Fig. 3g), and predicted a strong interaction between the proteins of AADAC and CES1, as well as the proteins of AADAC and CES2. Both CES1 and CES2 are involved in the metabolism of xenobiotics.

### AADAC expression and patient survival

**Fig. 4:**
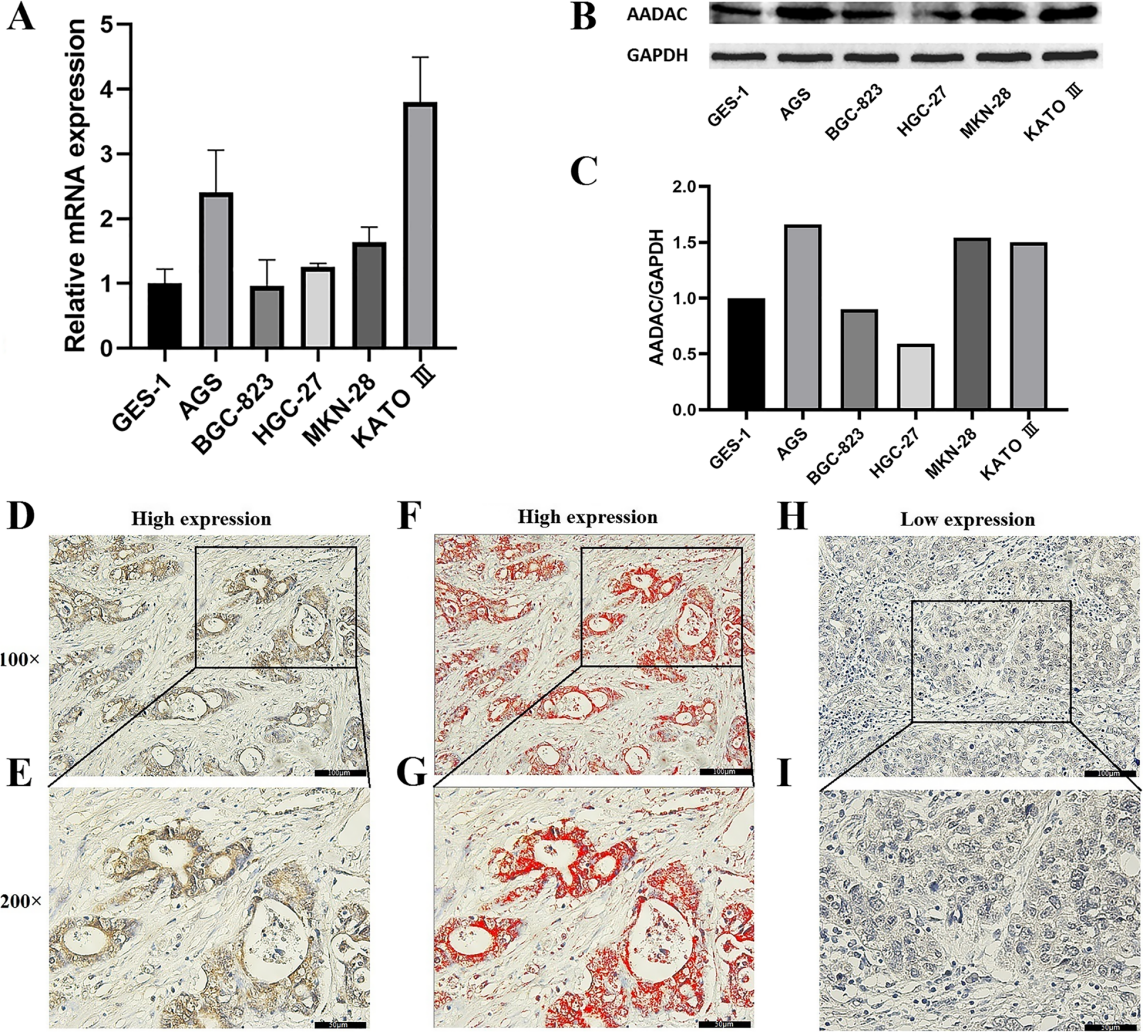
Expression of AADAC. **a** The mRNA expression of AADAC in 6 cell lines. **b** The protein expression of AADAC in 6 cell lines. **c** Amounts of AADAC protein determined by densitometry of protein bands. GAPDH was the loading control. **d-e** Immunohistochemical (IHC) staining of high expression of AADAC in Borrmann type III AGC. **f-g** Image processed by Image-Pro Plus version software. **h-i** IHC staining of low expression of AADAC in Borrmann type III AGC. Western blot (WB) images had been croppedand and the full length original blots were included in the Supplementary file 4

The mRNA and protein levels of AADAC from several GC cell lines and the normal gastric epithelial cell line GES-1 were detected by qRT-PCR and WB. We found that AADAC mRNA was highly expressed in KATO III and AGS cells and expressed at low levels in BGC-823 and GES-1 cells (Fig. 4a). The AADAC protein was highly expressed in MKN-28, AGS and KATO III cells and expressed at low levels in BGC-823, HGC-27 and GES-1 cells (Fig. 4b, c).

To further confirm AADAC expression in GC samples, 152 patients with Borrmann type III AGC were randomly selected for immunohistochemical staining according to the postoperative pathological report. The age of the patients was 23-81 years (median age was 60 years). There were 113 male patients (74.3%) and 39 female patients (25.7%). The number of patients with pTNM stages I, II, and III was 11 (7.2%), 58 (38.2%) and 83 (54.6%), respectively (Supplementary file 3).

The expression of AADAC was mainly observed in the membrane of GC cells (Fig. 4d, e, h, i). In addition, Image-Pro Plus software can digitally express image information after immunohistochemical staining to effectively avoid the influence of subjective factors and facilitate further statistical analysis (Fig. 4f, g). The results were quantified as the positive area/total area of immune markers, and 5.0% was defined as the cutoff value. Eighty-eight GC patients with an area ratio ≤5.0% were defined as low AADAC expression patients, and 64 GC patients with an area ratio >5.0% were defined as high AADAC expression patients. The mean±standard deviation of area ratio in low and high expression groups were 0.0269±0.0114 and 0.0682±0.0161, respectively. In addition, the area ratio of the low AADAC expression group was lower and concentrated near the mean, while the area ratio of high AADAC expression group was more discrete.

**Fig. 5:**
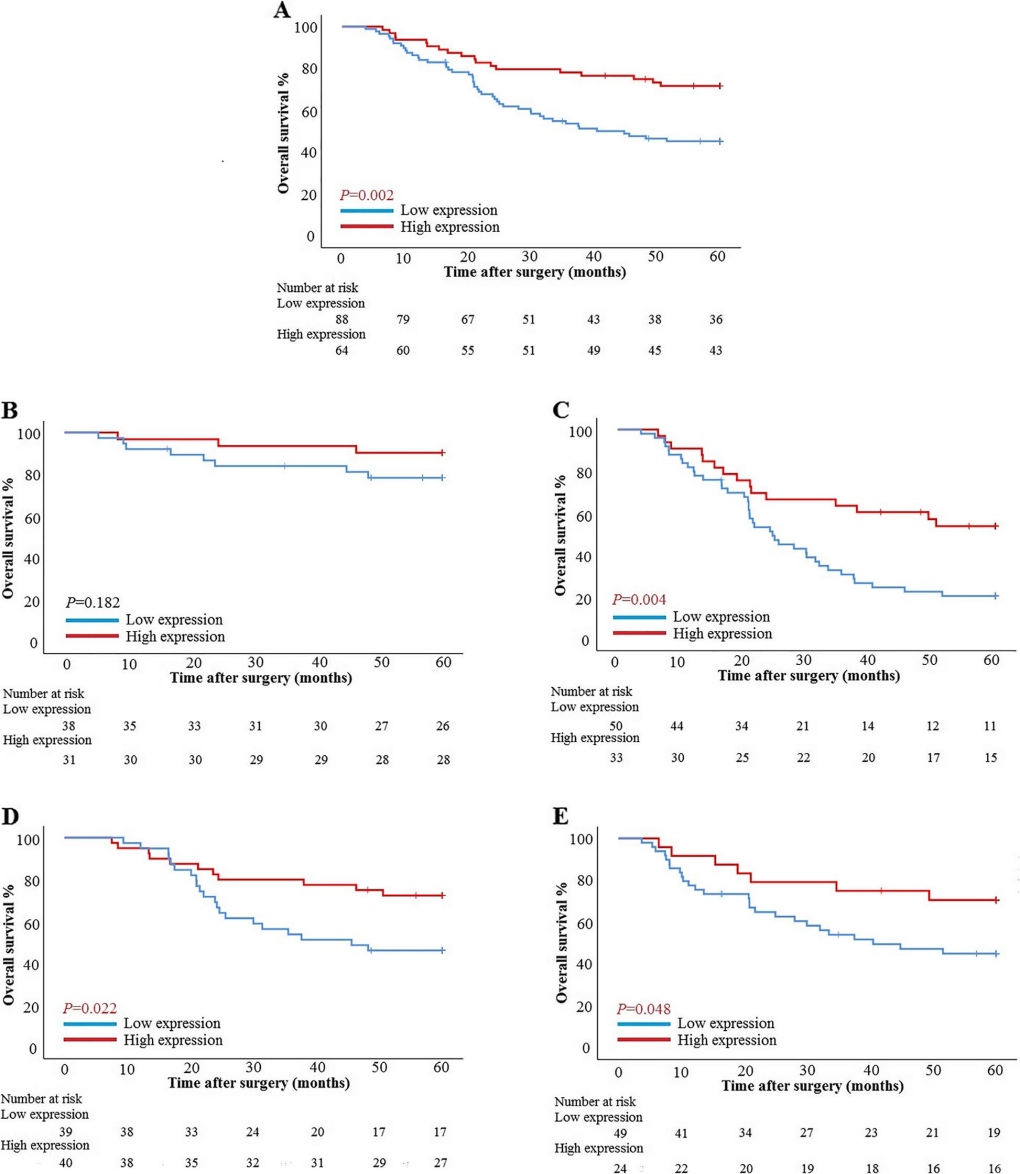
Kaplan–Meier curves of overall survival. **a** AADAC expression in all patients. **b** AADAC expression in patients with stage I-II disease. **c** AADAC expression in patients with stage III disease. **d** AADAC expression in patients aged ≤60 years. **e** AADAC expression in patients aged >60 years

The OS of patients with low expression of AADAC was 37.82 (95% CI: 33.250-42.399) months, and the 5-year OS rate was 43.1%. The OS of patients with high expression of AADAC was 49.64 (95% CI: 45.188-54.087) months, and the 5-year OS rate was 71.7%. There was a significant difference in OS between the two groups (*P* = 0.002) (Fig. 5a). According to pTNM stage, there was no significant difference in OS between patients with low expression of AADAC and patients with high expression of AADAC in stage I-II (*P* = 0.182). In stage III patients, there was a significant difference in OS between the two groups (OS: 29.94 months vs. 42.92 months, *P* = 0.004; HR: 0.420, 95% CI: 0.230-0.765) (Fig. 5b, c). The chi-square analysis showed that the expression of AADAC was statistically correlated with the age of Borrmann type III AGC patients (*P* = 0.027) (Table. 4). Subgroup analysis showed that there were significant differences in OS between the two groups with high and low expression of AADAC in different age groups (*P* = 0.022 and *P* = 0.048) (Fig. 5d, e).

**Table 4:** Chi-square test analysis of the connection between AADAC expression and clinicopathological features

**Characteristics**	**Low expression (*n *= 88)**	**High expression (*n *= 64)**	***p* value**
Sex			0.874
Male	65 (73.9)	48 (75.0)	
Female	23 (26.1)	16 (25.0)	
Age (years)			**0.027**
≤60	39 (44.3)	40 (62.5)	
>60	49 (55.7)	24 (37.5)	
BMI (kg/m^2^)			0.787
≤24	69 (78.4)	49 (76.6)	
>24	19 (21.6)	15 (23.4)	
Tumor diameter (mm)			0.809
≤50	45 (51.1)	34 (53.1)	
>50	43 (48.9)	30 (46.9)	
CEA			0.499
≤5ng/ml	72 (81.8)	55 (85.9)	
>5ng/ml	16 (18.2)	9 (14.1)	
CA19-9			0.885
≤37U/ml	75 (85.2)	54 (84.4)	
>37U/ml	13 (14.8)	10 (15.6)	
Tumor location			0.415
Middle and Upper third	22 (25.0)	21 (32.8)	
Lower third	65 (73.9)	43 (67.2)	
Entire stomach	1 (1.1)	0 (0)	
Histological type			0.583
Well to moderately differentialed	17 (19.3)	12 (18.8)	
Poor differentialed	36 (40.9)	31 (48.4)	
Signet ring cell	9 (10.2)	3 (4.7)	
Mucinous	26 (29.6)	18 (28.1)	
pTNM stage			0.813
I	6 (6.8)	5 (7.8)	
II	32 (36.4)	26 (40.6)	
III	50 (56.8)	33 (51.6)	
Vascular infiltration			0.405
Yes	26 (29.5)	23 (35.9)	
No	62 (70.5)	41 (64.1)	
Nerve infiltration			0.288
Yes	42 (47.7)	25 (39.1)	
No	46 (52.3)	39 (60.9)	
Postoperative chemotherapy			**0.038**
Yes	28 (31.8)	31 (48.4)	
No	60 (68.2)	33 ( 51.6)	
HER2 expression			0.403
Negative or IHC 1+	68 (77.9)	53 (82.8)	
IHC 2+ or IHC 3+	20 (22.1)	11 (17.2)	

*BMI* body mass index, *CEA* carcinoembryonic antigen, *CA19-9* carbohydrate antigen 19-9, *IHC* immunohistochemical

CEA and CA19-9 were according to the tumor marker examination. Tumor location, histological type, pTNM stage, vascular infiltration ,nerve infiltration and HER2 expression were according to the postoperative pathology report. Statistically significant *P* values are in bold (*P*<0.05)

**Fig. 6:**
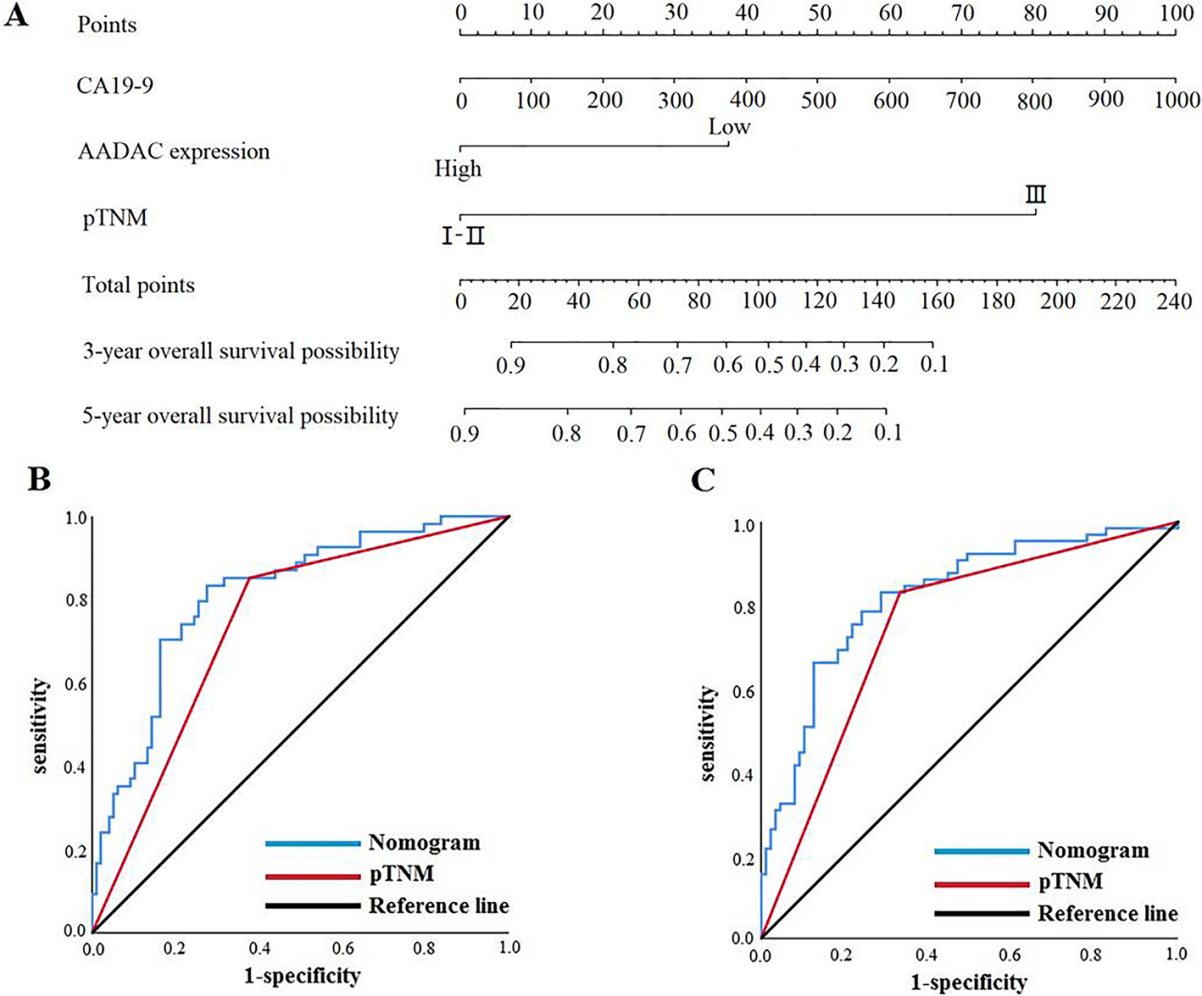
Nomogram models predicting the overall survival and disease-free survival of patients. **a** Nomogram model predicting the 3- and 5-year overall survival of patients with Borrmann type III AGC. **b** ROC curve of the nomogram model predicting the 3-year overall survival of patients. **c** ROC curve of the nomogram model predicting the 5-year overall survival of patients. ROC: receiver operating characteristic, CA19-9: carbohydrate antigen 19-9

Univariate and multivariate analyses based on the Cox proportional hazards regression model showed that CA19-9, pTNM stage and AADAC expression were independent risk factors associated with OS in patients with Borrmann type III AGC (Table 5). Furthermore, we combined the independent risk factors related to OS to construct a nomogram to evaluate the prognosis of patients (Fig. 6a). For predicting the OS of patients within 3 and 5 years after radical resection, the areas under the curves (AUCs) of the nomogram models were both greater than those of pTNM stage alone, 0.812 (95% CI: 0.742-0.883) and 0.821 (95% CI: 0.753-0.889) vs. 0.737 (95% CI: 0.656-0.818) and 0.759 (95% CI: 0.669-0.828), respectively. The sensitivity was 83.3% and 83.1%, and the specificity was 72.4% and 71.3%, respectively (Fig. 6b, c).

**Table 5:** Univariate and multivariate analyses of overall survival

**Characteristics**	**Univariate analysis**		**Multivariate analysis**	
	**HR (95% CI)**	***P* value**	**HR (95% CI)**	***P* value**
Sex				
Male	1[reference]			
Female	0.823 (0.462-1.465)	0.507		
Age (years)	1.020 (0.993-1.049)	0.147	-	-
BMI	0.956 (0.871-1.048)	0.336	-	-
CEA (ng/ml)	1.009 (1.000-1.017)	**0.038**	1.005 (0.996-1.014)	0.287
CA19-9 (U/ml)	1.003 (1.001-1.004)	**<0.001**	1.002 (1.001-1.004)	**0.003**
Tumor location				
Middle and Upper third	1[reference]			
Lower third	1.033 (0.599-1.782)	0.907		
Entire stomach	3.732 (0.492-28.304)	0.203		
Histological type				
Well to moderately differentialed	1[reference]		1[reference]	
Poor differentialed	1.441 (0.675-3.077)	0.345	1.282 (0.579-2.834)	0.540
Signet ring cell	1.014 (0.312-3.294)	0.981	0.547 (0.164-1.829)	0.327
Mucinous	2.473 (1.157-5.284)	**0.019**	2.005 (0.917-4.383)	0.081
Tumor size (mm)	1.006 (0.995-1.018)	0.295	-	-
pTNM stage				
I-II	1[reference]		1[reference]	
III	5.964 (3.108-11.445)	**<0.001**	4.388 (2.029-9.488)	**<0.001**
AADAC				
Low expression	1[reference]		1[reference]	
High expression	0.427 (0.248-0.737)	**0.002**	0.413 (0.235-0.727)	**0.002**
mLNR	28.014 (10.216-76.816)	**<0.001**	2.774 (0.554-13.895)	0.215
Vascular infiltration				
No	1[reference]		1[reference]	
Yes	1.673 (1.021-2.742)	**0.041**	1.083 (0.617-1.899)	0.782
Nerve infiltration				
No	1[reference]			
Yes	1.412 (0.860-2.320)	0.173		
Postoperative chemotherapy				
Yes	1[reference]			
No	1.082 (0.656-1.784)	0.757		

*HR* hazard ratio, *CI* confidence interval, *BMI* body mass index, *CEA* carcinoembryonic antigen, *CA19-9* carbohydrate antigen19-9, *mLNR* metastatic lymph node ratio

CEA and CA19-9 were according to the tumor marker examination. Tumor location, pTNM stage, histological type, mLNR, vascular infiltration and nerve infiltration were according to the postoperative pathology report. Statistically significant *P* values are in bold (*P*<0.05)

**Fig. 7:**
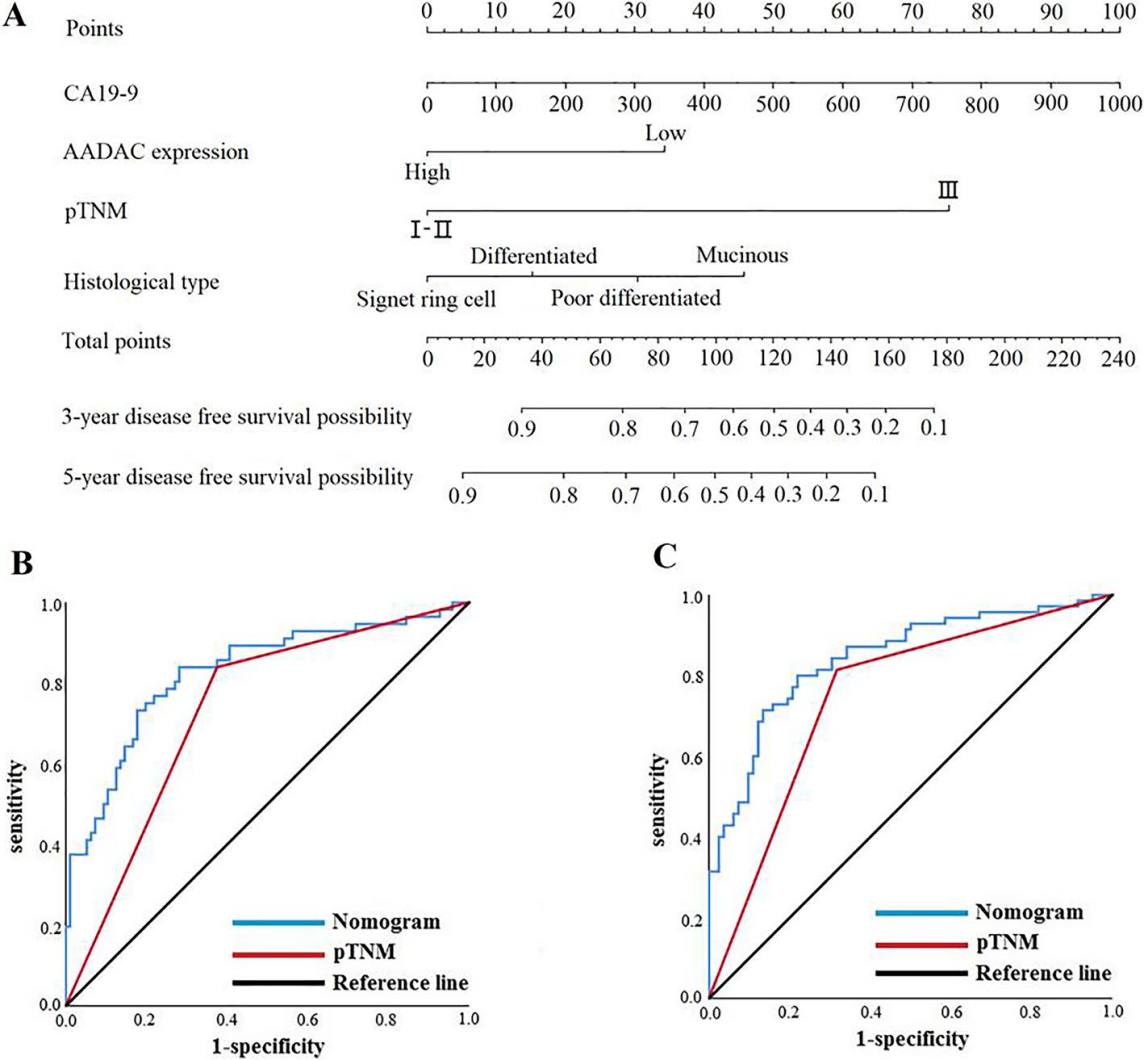
**a** Nomogram models predicting the disease free survival of patients a Nomogram model predicting the 3- and 5-year disease-free survival of patients with Borrmann type III AGC. **b** ROC curve of the nomogram model predicting the 3-year disease-free survival of patients. **c** ROC curve of the nomogram model predicting the 5-year disease-free survival of patients. ROC: receiver operating characteristic, CA19-9: carbohydrate antigen 19-9

Univariate and multivariate analyses based on the Cox proportional hazards regression model showed that CA19-9, histological type, pTNM stage and AADAC expression were independent risk factors associated with DFS in patients with Borrmann type III AGC (Table 6). Furthermore, we combined the independent risk factors related to DFS to construct a nomogram to evaluate the prognosis of patients (Fig. 7a). For predicting DFS within 3 and 5 years after radical resection, the AUCs of the nomogram models were both greater than those of pTNM stage alone, which were 0.826 (95% CI: 0.755-0.898) and 0.844 (95% CI: 0.780-0.907) vs. 0.732 (95% CI: 0.650-0.814) and 0.749 (95% CI: 0.669-0.828), respectively. The sensitivity was 83.9% and 80.0%, and the specificity was 71.9% and 78.0%, respectively (Fig. 7b, c).

**Table 6:** Univariate and multivariate analyses of disease-free survival

Characteristics	Univariate analysis		Multivariate analysis	
	HR (95% CI)	*P* value	HR (95% CI)	*P* value
Sex				
Male	1[reference]			
Female	0.878 (0.509-1.517)	0.642		
Age (years)	1.017 (0.991-1.044)	0.211	-	-
BMI	0.978 (0.895-1.068)	0.620	-	-
CEA (ng/ml)	1.008 (1.000-1.017)	0.051	-	-
CA19-9 (U/ml)	1.002 (1.001-1.004)	**<0.001**	1.002 (1.001-1.003)	**0.005**
Tumor location				
Middle and Upper third	1[reference]			
Lower third	0.923 (0.553-1.542)	0.759		
Entire stomach	3.050 (0.406-22.919)	0.279		
Histological type				
Well to moderately differentialed	1[reference]		1[reference]	
Poor differentialed	1.494 (0.703-3.178)	0.297	1.345 (0.619-2.922)	0.453
Signet ring cell	1.020 (0.314-3.312)	0.974	0.556 (0.168-1.835)	0.335
Mucinous	2.974 (1.410-6.272)	**0.004**	2.508 (1.167-5.389)	**0.019**
Tumor size (mm)	1.008 (0.997-1.019)	0.163	-	-
pTNM stage				
I-II	1[reference]		1[reference]	
III	5.453 (2.973-10.001)	**<0.001**	4.151 (2.011-8.570)	**<0.001**
AADAC				
Low expression	1[reference]		1[reference]	
High expression	0.469 (0.281-0.783)	**0.004**	0.432 (0.255-0.731)	**0.002**
mLNR	30.128 (11.074-81.966)	**<0.001**	3.113 (0.680-14.238)	0.143
Vascular infiltration				
No	1[reference]			
Yes	1.598 (0.990-2.581)	0.055		
Nerve infiltration				
No	1[reference]			
Yes	1.286 (0.801-2.065)	0.299		
Postoperative chemotherapy				
Yes	1[reference]	0.990		
No	0.997 (0.618-1.609)			

*HR* hazard ratio, *CI* confidence interval, *BMI* body mass index, *CEA* carcinoembryonic antigen, *CA19-9* carbohydrate antigen19-9, *mLNR* metastatic lymph node ratio

CEA and CA19-9 were according to the tumor marker examination. Tumor location, pTNM stage, histological type, mLNR, vascular infiltration and nerve infiltration were according to the postoperative pathology report. Statistically significant *P* values are in bold (*P*<0.05)

## Discussion

In China, more than 80% of GC patients are diagnosed with AGC upon hospital admission [3]. This is because most patients do not have obvious symptoms, such as dysphagia, vomiting or abdominal pain, in the early stages, which limits the early prevention and treatment of cancer in developing countries. Except for a few cases, AGC patients often have poor prognoses and relatively short survival times. Since 1926, Borrmann classification based on the macroscopic characteristics of GC has been proposed and widely used to evaluate the prognosis of AGC patients [4, 22]. Previous studies have shown that Borrmann type III is the most common macroscopic type of AGC, accounting for 40.6%–58.9%, and it has a 5-year survival rate of 46.0%-51.6%, with a prognosis between type I-II and type IV (5, 6). Previous studies have differed in the definition of high-risk Borrmann type III AGC. Yamashita et al. [23] considered that for patients with pTNM stage II/III, giant Borrmann type III AGC (≥8 cm) had similar sensitivity to postoperative adjuvant chemotherapy with Borrmann type IV AGC; Both belonged to the high-risk macroscopic type, and the 5-year survival rate was 35.7%. However, other studies found that patients with vascular infiltration Borrmann type III had the same poor prognosis as type IV, with a 5-year survival rate of 16.4%–19.4% [8, 24]. The reason for this divergence is the high heterogeneity of GC. Although GC is traditionally regarded as a single disease, GC is highly heterogeneous from the perspective of morphology and molecular science. This characteristic of GC is considered to be the main obstacle to its effective diagnosis and successful molecular-driven therapy [25]. Although the classifications [26] based on GC molecular characteristics effectively reduce this heterogeneity, there is no effective method to break through the heterogeneity of Borrmann type III AGC. Survival time is the most intuitive embodiment of the prognosis of patients with GC. We hope to group Borrmann type III AGC according to the significant difference in survival time to screen for its prognostic genes. We found by mRNA sequencing that AADAC is a prognostic differential gene of Borrmann type III AGC. In this study, we explored whether AADAC could be used as a new effective prognostic index to judge high-risk Borrmann type III AGC patients, guide their perioperative treatment and improve their prognosis.

AADAC is a member of the serine esterase superfamily and is expressed mainly in the liver and gastrointestinal tract, which are involved in the metabolism of clinical drugs such as flutamide, phenacetin, and rifamycins [27, 28]. AADAC deacetylates a variety of arylacetamide substrates in the liver, including xenobiotic compounds and carcinogens, and converts them to primary arylamide compounds [29]. In addition, AADAC also displays cellular triglyceride lipase activity in the liver, increases the levels of intracellular fatty acids by hydrolyzing triglycerides and plays a role in very low-density lipoprotein assembly [30]. We hope to determine the clinical significance of AADAC in patients with Borrmann type III AGC by analyzing its mRNA and protein expression levels and exploring its role in the progression of GC.

In this study, we found that AADAC showed oncogenic effects in the TCGA-STAD dataset and anti-oncogene effects in the GSE15459 dataset and our HMU-GC cohort. We tried to determine the function of AADAC in patients with different datasets through KEGG and GO gene enrichment analysis. In the TCGA-STAD dataset, we found that AADAC and its related genes may play an important role in biofilm synthesis and cytoskeleton formation through KEGG and GO analysis. In fact, cancer is essentially a disorder of cell growth and proliferation that requires a large amount of cellular biomass, such as nucleic acids, proteins and lipids. KEGG analysis showed that AADAC expressed triglyceride lipase and amino acid hydrolase activities in GC. This biological function provides raw materials such as fatty acids and amino acids for the proliferation of cancer cells and promotes biofilm synthesis and cytoskeleton formation [30, 31]. Therefore, we believe that in the European and American patient populations of the TCGA-STAD dataset, AADAC provides raw biomass and energy for the rapid proliferation of cancer cells by increasing the levels of fatty acids and amino acids in cancer cells to promote cancer progression. In the GSE15459 dataset, we found that AADAC and its related genes may play an important role in the degradation of chemical carcinogens and inhibit cell migration by KEGG and GO analysis. AADAC expresses serine lipase activity and participates in the metabolism of a variety of endogenous and exogenous substances and clinical drugs. In the metabolism of chemical carcinogens, benzo [a] pyrene (BAP) can be metabolized by AADAC and its related genes and then transformed into DNA adducts [32]. Wei et al. found that BAP promoted the proliferation and metastasis of GC cells by up-regulating the expression of matrix metalloproteinase-9 and c-myc and activating aromatic hydrocarbon receptors and the ERK pathway [33]. The above results show that AADAC mainly expresses serine lipase activity in the Asian patient population of the GSE15459 dataset and maintains gastrointestinal epithelial homeostasis by catabolizing exogenous chemical carcinogens to inhibit the progression of GC. Similarly, in the HMU-GC cohort, we found that by KEGG and GO analysis, AADAC and its related genes are critical in the metabolism of carbohydrate, lipid and energy. Metabolic changes are considered to be a marker of cell malignant transformation [34]. The specific adaptation in the anabolic pathway provides the raw materials needed to produce nucleic acids, proteins and lipids for the rapid proliferation of cancer cells, promoting the formation of biomass [35, 36]. AADAC mainly participated in the biomass metabolism of cancer cells in the HMU-GC cohort, which may play a tumor inhibitory role by limiting the synthesis of essential substances for tumor cell proliferation. It can explain why patients with high expression of AADAC are better for OS in our hospital cohort. In addition, the protein-protein interaction networks also showed that AADAC and its related genes such as CES1 and CES2 are jointly involved in the metabolism of xenobiotics [29]. Our study found that AADAC mainly participates in the metabolic process of tumor cells as a member of the serine lipase superfamily. It maintains gastrointestinal epithelial homeostasis and inhibits the progress of GC by catabolizing exogenous chemical carcinogens and inhibiting the synthesis of tumor essential biomass.

We found that AADAC was an oncogene in the TCGA-STAD cohort dominated by European and American populations and an anti-oncogene in the GSE15459 cohort dominated by Asian populations. All patients included in our HMU-GC cohort were Chinese, and the low expression of AADAC at the mRNA and protein levels was associated with poor 5-year OS, which was the same trend as that of the GSE15459 cohort. This difference may be related to the genetic polymorphisms of AADAC. Drug-metabolizing enzymes are often affected by genetic polymorphisms, thereby changing protein expression or catalytic activity [37]. As a metabolic enzyme of many drugs, AADAC is known to have three alleles. Shimizu et al. [38] found that AADAC*1 (wild-type) and AADAC*2 alleles are distributed in European American, African American, Japanese and Korean populations. The AADAC*3 allele is only distributed in European American and African American populations but not in Japanese and Korean populations. Compared with the other two alleles, the protein expression and enzyme activity of AADAC*3 decreased significantly. This conclusion confirms that there are differences in the protein expression and enzyme activity of AADAC between European, American and Asian populations, which is consistent with our results. AADAC may show weak serine lipase activity and strong triglyceride lipase activity in European and American populations, mainly by promoting biofilm synthesis to promote the progression of GC. AADAC shows strong serine lipase activity in the Asian population, maintains gastrointestinal epithelial homeostasis by neutralizing exogenous chemical carcinogens and inhibits the progression of GC. This difference may be related to different genetic and environmental factors in European, American and Asian populations. Because of differences in eating habits, European and American populations are more likely to be exposed to chemical carcinogens such as heterocyclic amines, resulting in changes in genetic polymorphisms [39].

We analyzed the relationship between AADAC expression and clinicopathological features and found that high AADAC expression was associated with young patients and metabolic subtype patients. Previous studies have shown that the metabolic activation of a variety of chemical carcinogens, such as polycyclic hydrocarbons, N-nitrosamines and aromatic amines, is age-dependent. Their metabolism is low in fetal tissue and decreases with age in adulthood [40]. This may be related to the decreased activity of metabolic enzymes in chemical carcinogens. In addition, there was evidence that the induction of rat hepatic O6-methylguanine-DNA methyltransferase activity by 2-acetylaminofluorene, a liver carcinogen requiring metabolism, will increase with age and then induce cancer. Our study confirmed that the expression of AADAC in patients with Borrmann type III AGC decreased with age, showing lower metabolic activity of chemical carcinogens and reduced ability to maintain gastrointestinal epithelial homeostasis. Our study found that AADAC was differentially expressed in four subtypes of GC (invasive, proliferative, metabolic and unstable), and the expression was the highest in the metabolic type. The research results of Zeng et al. [41] are consistent with ours. They found that metabolic subtypes are mainly related to the positive regulation of hydrolase activity, catalytic activity, cell division, and cell cycle phase. In addition, they found that the p53 signaling pathway is enriched in metabolic subtypes and plays an important role. The mutation of genes in the p53 signaling pathway is the most common genetic change in cancers [42]. This provides ideas for us to study the detailed mechanism of AADAC affecting the occurrence and development of GC.

In this study, we verified the protein and mRNA expression of AADAC in GC cell lines. We found that the expression of AADAC in gastric epithelial cell line (GES-1) was lower than that in some GC cell lines. In normal tissues, proto oncogenes are usually in a state of low or no expression [43]. However, under some conditions, such as repeated viral infection, chemical carcinogens or radiation, proto oncogenes can be abnormally activated and transformed into oncogenes to induce abnormal cell proliferation [44]. Our study found that AADAC plays a role in the metabolism of exogenous chemicals. When normal gastric epithelial cells are affected by chemical carcinogens, AADAC is activated and catabolizes carcinogens. This can explain its low expression level in normal gastric epithelial cells. In addition, we found that AADAC was highly expressed in highly differentiated GC cell lines such as AGS and MKN-28, while it was expressed at low levels in poorly differentiated GC cell lines such as BGC-823 and HGC-27. The expression level of AADAC was related to the degree of differentiation of GC tissues, but not to the degree of malignancy of GC tissues. Our study found that AADAC maintains gastrointestinal epithelial homeostasis through the catabolism of exogenous chemical carcinogens. GC tissues with a high expression of AADAC more easily maintain epithelial homeostasis, which shows a higher degree of differentiation. Besides, we found that the expression level of AADAC was high in the Kato III GC cell line with signet ring cell carcinoma (SRC). SRC, a special pathological type, has a good prognosis in EGC and a poor prognosis in AGC [45, 46]. It may suggest that driver mutations controlling the metastatic potential of SRC can occur late in GC progression. Studying the effect of AADAC expression on the prognosis of SRC may provide a new idea for studying the mechanism of this special biological behavior.

In clinical practice, medical experts gradually found that TNM stage based on postoperative pathology provides effective but incomplete information for treatment. The prognosis of patients’ at the same stage showed significant individual differences. Yin et al. [47] constructed a nomogram based on CD144 and pTNM staging to predict the prognosis of stage III GC patients. Therefore, the prediction models based on the combination of molecular biomarkers and clinicopathological features have the advantages of more accurate and individualized evaluation of patients prognosis and reducing the differences caused by heterogeneity. Based on the Cox hazards regression model, we found that CA19-9, pTNM stage and AADAC expression were independent risk factors associated with the OS of patients with Borrmann type III AGC, and CA19-9, histological type, pTNM stage and AADAC expression were independent risk factors associated with the DFS of patients with Borrmann type III AGC. Then, we constructed nomogram models to predict the prognosis of patients with Borrmann type III AGC. ROC curves analysis showed that the AUCs for predicting 3-year and 5-year OS were 0.812 and 0.821, respectively. The sensitivity was 83.3% and 83.1%, and the specificity was 72.4% and 71.3%, respectively. The AUCs for predicting 3-year and 5-year DFS were 0.826 and 0.844, respectively. The sensitivity was 83.9% and 80.0%, and the specificity was 71.9% and 78.0%, respectively. We found that the nomogram models were better than conventional pTNM stage alone in predicting the prognosis of patients with Borrmann type III AGC within 3-year and 5-year after radical resection. The prediction models constructed by the molecular biomarkers and clinicopathological features can effectively evaluate the prognosis of patients with Borrmann type III AGC, which is worthy of further validation and promotion in clinical practice.

Whether AADAC can be used as a potential therapeutic target of Borrmann type III AGC is our next research direction. Lei et al. [48] found that GC patients with metabolic subtypes were more sensitive to 5-fluorouracil treatment. Since the high expression of AADAC is associated with GC patients with metabolic subtypes, we hope to provide new treatment strategies for GC patients by studying the sensitivity of AADAC to chemotherapeutic drugs in the future. In addition, the regulation of the lipolysis pathway may represent a new approach to human cancer treatment. Previous studies have found that the loss of rate-limiting enzymes for triglyceride hydrolysis is associated with human cancer and mouse pulmonary neoplasia [49]. AADAC, as a triglyceride hydrolase, will promote the progression of GC in European and American populations. In the future, inhibiting the expression of AADAC to control the proliferation of cancer cells will become a potential treatment strategy for GC. In view of the functional differences of AADAC in different populations, we hope to explore different treatment strategies for different races.

There were some limitations in this study. First, this was a single-center study with a small sample size. In the future, it will be necessary to expand the sample size through multicenter research to further verify the prognostic significance of AADAC for GC patients. Second, this study only verified the mRNA expression level of AADAC in different populations through public databases. Exploring the expression difference of AADAC protein levels in different populations is a further research direction. Third, this study did not elaborate on the detailed molecular mechanisms of AADAC inhibiting the development of GC and its application in the treatment of GC. More basic experiments are needed in the future to study this detailed molecular mechanism and whether AADAC can be used as a new therapeutic target for GC.

## Conclusions

Our results showed that AADAC overexpression is significantly associated with the good prognosis of Borrmann type III AGC and inhibits the progression of GC. AADAC showed significant differences in prognosis and function in different populations. AADAC can be used as an independent prognostic factor in patients with Borrmann type III AGC.

## Supplementary Information

**Additional file 1:** **Supplementary file 1.** Baseline characteristics of the patients in HMU-GC cohort. **Supplementary file 2.** The expression of AADAC in TCGA-STAD dataset. **Supplementary file 3.** Baseline characteristics of the patients. **Supplementary file 4.** The full length original Western blot (WB) images. 

## Data Availability

The datasets generated and/or analysed during the current study are available in the [Gastric Cancer Information Management System v1.2 of Harbin Medical University Cancer Hospital] repository, [Copyright No. 2013SR087424, http://www.sgihmu.com].

## Abbreviations

GC: Gastric cancer
AGC: Advanced gastric cancer
EGC: Early gastric cancer
DEGs: Differentially expressed genes
OS: Overall survival
DFS: disease-free survival
TCGA: The Cancer Genome Atlas
GEO: Gene Expression Omnibus
qRT-PCR: quantitative real-time reverse-transcription polymerase chain reaction
WB: Western blot
IHC: Immunohistochemical
AADAC: Arylacetamide deacetylase
HMU: Harbin Medical University
pTNM: postoperative Tumor-node-metastasis
HR: Hazard ratio
CI: Confidence interval
ROC: Receiver operating characteristic
AUCs: Area under the curves
AJCC: American Joint Committee on Cancer
BMI: Body mass index
mLNR: metastatic Lymph node ratio
CA: Carbohydrate antigen
CEA: Carcinoembryonic antigen
CT: Computed tomography
PET: Positron emission tomography
XELOX: Oxaliplatin +capecitabine
SOX: oxaliplatin +S-1
KEGG: Kyoto Encyclopedia of Genes and Genomes
GO: Gene Ontology
PPI: Protein-protein interaction
SDS-PAGE: sulfate-polyacrylamide gels
PVDF: polyvinylidene fluoridend
TBS: Tris-buffered saline
DAB: diaminobenzidine
BAP: benzo [a] pyrene
SRC: signet ring cell carcinoma
