# Supplementary material for: Impact of AADAC gene expression on prognosis in patients with Borrmann type III advanced gastric cancer
Source: BMC Cancer. 2022 Jun 9;22:635. doi: 10.1186/s12885-022-09594-1 (PMC9178847; doi:10.1186/s12885-022-09594-1)

Supplementary Material

Supplementary file 1. Baseline characteristics of the patients in HMU-GC cohort.

Characteristics	A (n = 11)	B (n = 14)	*P* value
Sex			0.250
Male	8 (72.7)	7 (50.0)	
Female	3 (27.3)	7 (50.0)	
Age (years)			0.302
≤60	4 (36.4)	8 (57.1)	
>60	7 (63.6)	6 (42.9)	
Tumor diameter (mm)			0.792
≤50	2 (18.1)	2 (14.3)	
>50	9 (75.0)	12 (85.7)	
Tumor location			0.897
Upper third or Middle third	5 (45.5)	6 (42.9)	
Lower third	6 (54.5)	8 (57.1)	
Resection			0.383
Partial gastrectomy	6 (54.5)	10 (71.4)	
Total gastrectomy	5 (45.5)	4 (28.6)	
Histological type			0.653
Differentiated	3 (27.3)	5 (35.7)	
Undifferentiated	8 (72.7)	9 (64.3)	
pTNM stage			1.000
I	0 (0.0)	0 (0.0)	
II	0 (0.0)	0 (0.0)	
III	11 (100.0)	14 (100.0)	
Vascular infiltration			0.135
No	3 (27.3)	8 (57.1)	
Yes	8 (72.7)	6 (42.9)	
Nerve infiltration			0.366
No	0 (0.0)	1 (7.1)	
Yes	11 (100.0)	13 (92.9)	
CEA			0.087
≤5	5 (45.5)	11 (27.3)	
>5	6 (54.5)	3 (72.7)	
CA19-9			0.095
≤37	8 (72.7)	13 (92.9)	

>37	3 (27.3)	1 (7.1)	
Postoperative chemotherapy			0.409
Yes	7 (58.4)	11 (30.8)	
No	4 (41.6)	3 (69.2)	

CEA: carcinoembryonic antigen; CA19-9: carbohydrate antigen 19-9.

CEA and CA19-9 were according to the tumor marker examination. Tumor location, histological type, pT stage, pN stage, pTNM stage, vascular infiltration and nerve infiltration were according to the postoperative pathology report. Statistically significant *P* values are in bold (*P*<0.05).

Supplementary file 2. The expression of AADAC in TCGA-STAD dataset.

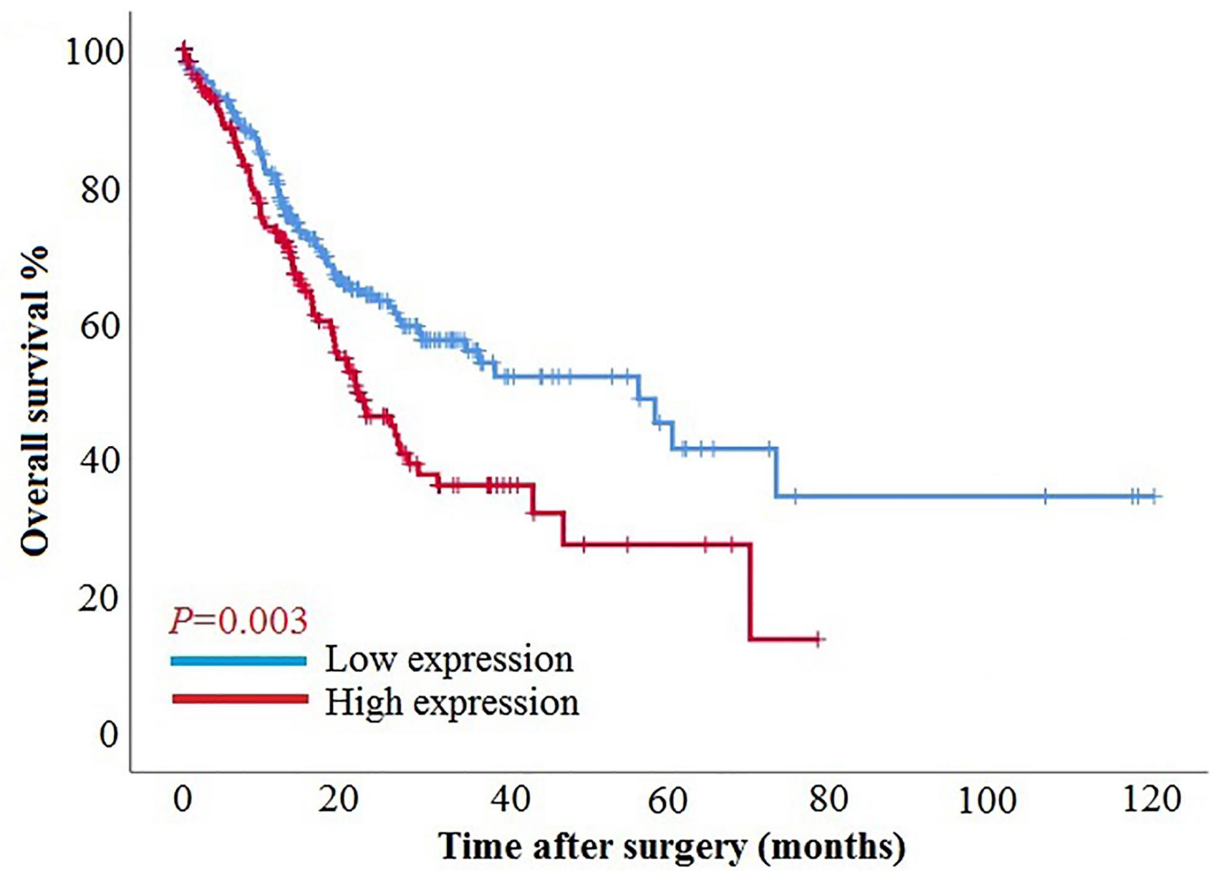

Supplementary file 3. Baseline characteristics of the patients

Characteristics	Cases（152）	Percentage(%)
Sex		
Male	113	74.3
Female	39	25.7
Age (years)		
≤60	79	52.0
>60	73	48.0
BMI(kg/m^2)		
≤24	118	77.6
>24	34	22.4
Tumor diameter (mm)		
≤50	79	52.0
>50	73	48.0
CEA		
≤5ng/ml	127	83.6
>5ng/ml	25	16.4
CA19-9		
≤37U/ml	129	84.9
>37U/ml	23	15.1
AADAC expression		
Low	88	57.9
High	64	42.1
Tumor location		
Middle and Upper third	43	28.2
Lower third	108	71.1
Entire stomach	1	0.7
Histological type		
Well to moderately differentialed	29	19.1
Poor differentialed	67	44.1
Signet ring cell	12	7.9
Mucinous	44	28.9
pT stage		
T2	23	15.1
T3	72	47.4
T4	57	37.5
pN stage		
N0	34	22.4

N1	46	30.3
N2	35	23.0
N3	37	24.3
pTNM stage		
I	11	7.2
II	58	38.2
III	83	54.6
Vascular infiltration		
Yes	49	32.2
No	103	67.8
Nerve infiltration		
Yes	81	53.3
No	71	46.7
Postoperative chemotherapy		
Yes	59	38.8
No	93	61.2

BMI: body mass index; CEA: carcinoembryonic antigen; CA19-9: carbohydrate antigen 19-9.

CEA and CA19-9 were according to the tumor marker examination. Tumor location, histological type, pT stage, pN stage, pTNM stage, vascular infiltration and nerve infiltration were according to the postoperative pathology report. Statistically significant *P* values are in bold ($P<0.05$).

Supplementary file 4. The full length original Western blot (WB) images

4.1 Western blot (WB) image of AADAC

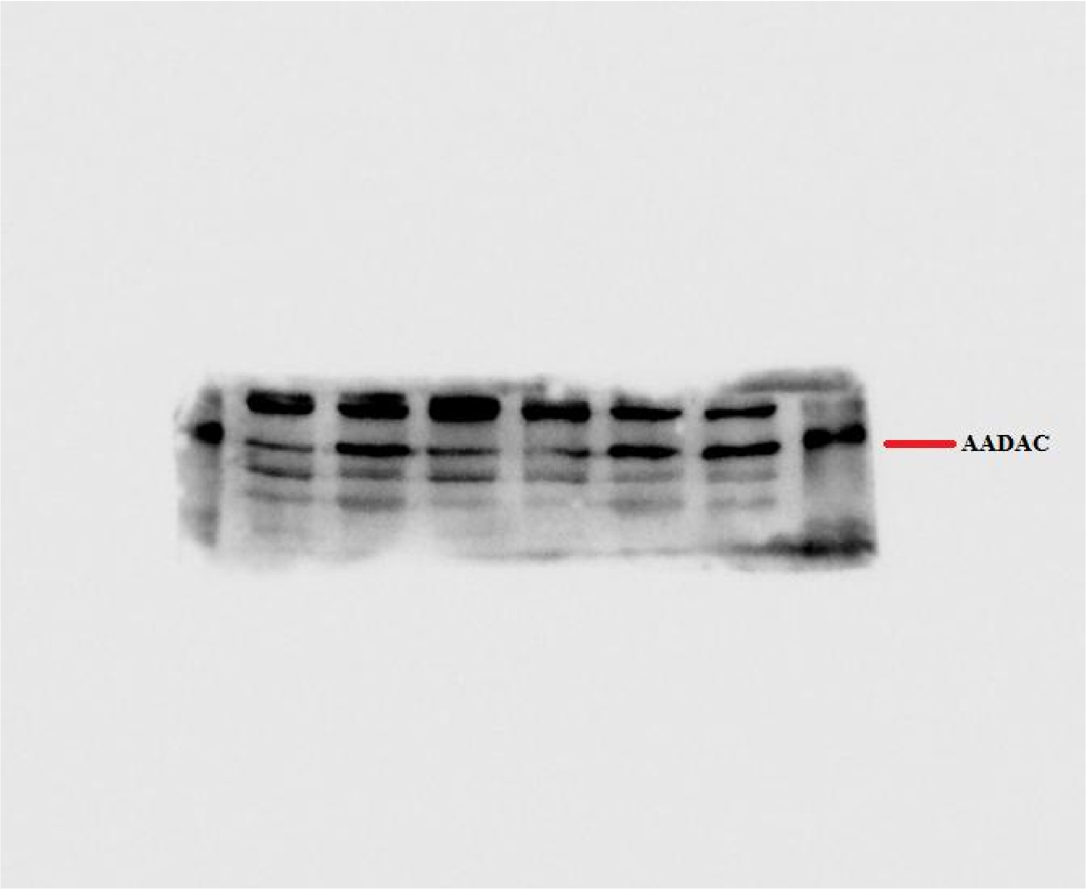

4.2 Western blot (WB) image of GAPDH

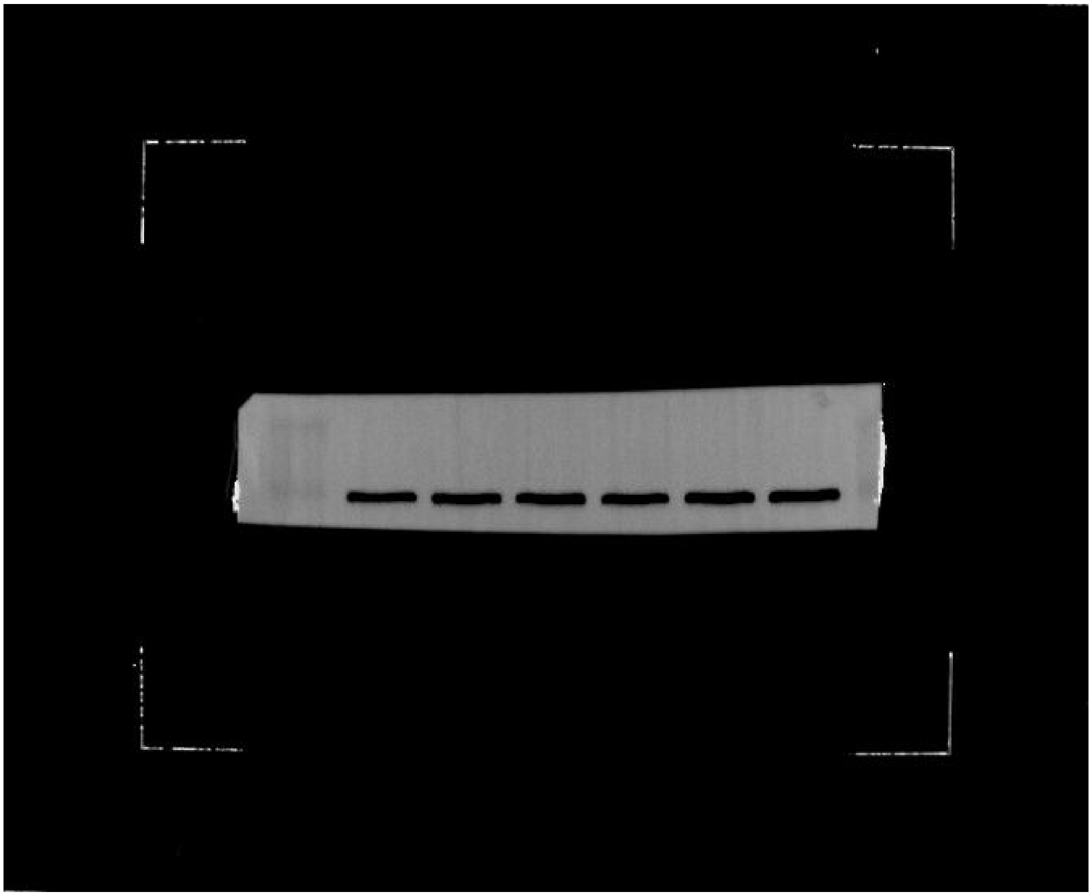

Supplement: Supplementary file 1 — Additional file 1: Supplementary file 1. Baseline characteristics of the patients in HMU-GC cohort. Supplementary file 2. The expression of AADAC in TCGA-STAD dataset. Supplementary file 3. Baseline characteristics of the patients. Supplementary file 4. The full length original Western blot (WB) images. [file 12885_2022_9594_MOESM1_ESM.pdf]
